# Pregnancy- and parenting-related barriers to receiving medication for opioid use disorder: A multi-paneled qualitative study of women in treatment, women who terminated treatment, and the professionals who serve them

**DOI:** 10.1177/17455057231224181

**Published:** 2024-02-16

**Authors:** Hannah B Apsley, Kristina Brant, Sarah Brothers, Eric Harrison, Emma Skogseth, Robert P Schwartz, Abenaa A Jones

**Affiliations:** 1 Department of Human Development and Family Studies, The Pennsylvania State University, University Park, PA, USA; 2 Department of Agricultural Economics, Sociology, and Education, The Pennsylvania State University, University Park, PA, USA; 3 Department of Sociology and Criminology, The Pennsylvania State University, University Park, PA, USA; 4 Friends Research Institute, Baltimore, MD, USA

**Keywords:** barriers, criminal justice, medication for opioid use disorder, parenting, pregnancy, qualitative, substance use, women

## Abstract

**Background:**

Women face unique barriers when seeking treatment for substance use disorders, often related to pregnancy and parenting.

**Objectives:**

This study adds to the extant literature by elucidating the pregnancy- and parenting-related barriers women face when initiating or continuing medication for opioid use disorder, specifically.

**Design:**

This study is based on qualitative semi-structured interviews.

**Methods:**

Three subgroups participated in semi-structured interviews regarding their experiences (N = 42): women with current or past opioid use disorders who have used or were presently using medication for opioid use disorder, professionals working in substance use disorder treatment programs, and criminal justice professionals.

**Results:**

Three parenting-related subthemes were identified: (1) insufficient access to childcare to navigate appointments and meetings, (2) fear of losing custody of, or access to, one’s children, and (3) prioritizing one’s children’s needs before one’s own. Three subthemes were identified with regard to pregnancy as a barrier: (1) hesitancy among physicians to prescribe medication for opioid use disorder for pregnant patients, (2) limited access to resources in rural areas, and (3) difficulty navigating a complex, decentralized health system.

**Conclusion:**

Systemic changes are needed to reduce pregnant and parenting women’s barriers to seeking medication for opioid use disorder. These include improved childcare support at both in-patient and outpatient treatment programs, which would assuage women’s barriers related to childcare, as well as their fears of losing access to their children if they spend time away from their children for treatment. An additional systemic improvement that may reduce barriers for these women is access to comprehensive, integrated care for their prenatal care, postpartum care, pediatric appointments, and appropriate substance use disorder treatment.

## Plain language summary

Pregnancy and parenting-related barriers to receiving medication for opioid use disorder: Interview themes from multiple perspectives

Women face unique barriers when seeking treatment for substance use disorders, often related to pregnancy and parenting. This study used one-on-one interviews to learn more about the pregnancy- and parenting-related barriers women face when initiating or continuing medication for opioid use disorder, specifically. Three different groups were interviewed: women with current or past opioid use disorders who have used or were presently using medication for opioid use disorder, professionals working in substance use disorder treatment programs, and criminal justice professionals (N = 42). Three parenting-related themes emerged from the interviews: (1) insufficient access to childcare to navigate appointments and meetings, (2) fear of losing custody of, or access to, one’s children, and (3) prioritizing one’s children’s needs before one’s own. Three pregnancy-related themes emerged from the interviews: (1) hesitancy among physicians to prescribe medication for opioid use disorder for pregnant patients, (2) limited access to resources in rural areas, and (3) difficulty navigating a complex health system. Systemic changes are needed to reduce pregnant and parenting women’s barriers to seeking medication for opioid use disorder. These include improved childcare support at treatment programs, which would assuage women’s barriers related to childcare, as well as their fears of losing access to their children if they spend time away from their children for treatment. An additional systemic improvement that may reduce barriers for these women is access to comprehensive, integrated care for their prenatal care, postpartum care, pediatric appointments, and appropriate substance use disorder treatment.

## Introduction

In 2021, an estimated 5.6 million adolescents and adults met the diagnostic criteria for opioid use disorder (OUD) in the United States.^1^ OUD carries high risks; in 2021, over 80,000 people in the United States died of an opioid-related overdose.^2^ Medications for opioid use disorder (MOUD), such as methadone and buprenorphine, are effective at reducing overdose deaths,^3^ improving quality of life generally,^4–6^ as well as in specific domains, such as mental health,^7^ reduced HIV risk behavior,^8^ and hepatitis C reinfection.^9^ However, in 2021, only 22.1% of individuals with OUD received medication for their opioid use.^1^

Extant research has identified barriers to receiving MOUD from the perspective of individuals with OUD^10,11^ and professionals involved in administering MOUD.^10–12^ From the patients’ perspectives, commonly reported barriers to receiving buprenorphine and naltrexone specifically include feeling stigmatized, not seeing the need for MOUD, perceiving that MOUD is not “real” recovery, the cost of the treatment, and long wait times to be seen by a provider.^13^ From the perspectives of physicians and other professionals, commonly reported barriers include holding stigma toward those with OUD and toward MOUD as a form of treatment, insufficient training and education regarding MOUD, insufficient time and resources to complete such training, low insurance reimbursement for MOUD provision, and concerns regarding MOUD diversion.^13^ Concerning methadone, patient-reported barriers to initiating or sustaining treatment are often logistical, such as burdensome travel to the clinic or pharmacy (often required daily), conflicts with one’s work schedule,^14^ and—similarly to the previously described barriers—long waiting lists, and lack of insurance or financial resources.^15^

However, people with OUD are not a homogeneous group. Women—specifically pregnant and parenting women—may have unique barriers to accessing MOUD. To date, prior work has compared the barriers that men and women face in seeking treatment for substance use disorders (SUDs) generally^16^ and has sought to categorize women’s barriers.^17^ Even though gender roles have become more equitable in recent decades, women still spend more time than men caring for their children.^18^ This persistent imbalance has important implications for treatment seeking. Only 5.5% of American SUD treatment facilities provide childcare,^19^ limiting the SUD treatment facilities that mothers may use if they do not already have access to stable childcare. In addition, mothers often report feeling added guilt for their substance use because of their children, and experiencing additional stigma from both their care providers and from family and friends.^20^ Women with children in their homes are also more likely to experience logistical barriers to seeking treatment, such as simply not having the time, when compared to women without children.^17^

Pregnant women also experience particular barriers to receiving treatment for SUDs. These barriers include fear of involvement of Child Protective Services (CPS) if a woman were to seek OUD treatment^21,22^ and stigma for having used substances while pregnant.^22^ Pregnant women also report experiencing structural and logistical barriers, such as being excluded from treatment services due to pregnancy and fear of losing eligibility for Medicaid in certain states following their child’s birth.^20^ In summation, both qualitative and quantitative studies have identified the internal, logistical, and structural barriers faced by pregnant and parenting women in seeking treatment for SUDs.

Prior work has generally assessed the barriers that pregnant and parenting women face in receiving SUD treatment generally, for a broad range of substances. However, there is limited work assessing the barriers this special population may experience when seeking or receiving MOUD for opioid use, specifically. These barriers are especially important to assess, considering that drug overdose mortality rates for pregnant and postpartum people increased by 81% from 2017 to 2021 and that the majority of these deaths were due to heroin, fentanyl, and other synthetic opioids.^23^ Schiff and colleagues^24^ employed qualitative semi-structured interviews with 26 recently postpartum women with OUD to assess barriers to receiving MOUD. Multiple themes emerged, including feeling a lack of autonomy when deciding to accept the medication, fear of causing harm to their infant, stigmatization by medical staff, fear of mandated reporting and loss of custody, and balancing treatment with challenging parenting responsibilities.

It is particularly important to continue building knowledge about the barriers pregnant and parenting women face in seeking MOUD for multiple reasons. Continued illicit opioid use puts pregnant women at risk of overdose and infection and increases risks to the fetus.^25,26^ If pregnant women attempt to abstain from substances and then experience a destabilization of their recovery, this recurrence of use can also harm both the pregnant person and the fetus.^27^ Use of a partial (e.g. buprenorphine) or full opioid agonist (e.g. methadone) as part of formal treatment is correlated with the best outcomes both for mothers’ OUD recovery, as well as infants’ health, when compared to continued use of illicit opioids.^28^ Even after the postpartum period, mothers’ successful treatment and recovery from SUDs has positive implications for the whole family system because substance use by parents has been associated with deleterious family outcomes, such as CPS involvement and children’s substance use.^29^

This study adds to previous work assessing the parenting and pregnancy-related barriers women with OUD face in seeking MOUD treatment. It builds upon prior work by triangulating the accounts of women who have used MOUD as part of an OUD treatment program, SUD treatment professionals, and criminal justice professionals. In so doing, we hope to identify barriers that may be possible points of intervention to increase pregnant and parenting women’s initiation and retention in MOUD treatment programs.

## Methods

### Participants and recruitment

Three subsamples of participants were recruited for this study. Sub-sample sizes were chosen in line with recent recommendations for achieving data saturation or redundancy.^30^ First, the study team recruited 20 women with lived experience using MOUD. To be eligible for the study, these women needed to meet the following inclusion criteria: (1) have previously used or currently use MOUD, (2) have a history of criminal justice involvement, (3) live in Pennsylvania, and (4) be 18 or older. These women were recruited through advertisements on social media, chain referrals, and recruitment flyers sent to MOUD treatment facilities in Pennsylvania.

Second, the study team recruited 10 criminal justice professionals who work with women with OUD. To be eligible, these professionals needed to meet the following inclusion criteria: (1) work with justice-involved women with OUD, (2) work in law enforcement, corrections, or the court system, (3) work in Pennsylvania, and (4) be 18 or older. These professionals were recruited through advertisements on social media, chain referrals, and direct phone calls to women’s prisons.

Third and finally, the study team recruited 12 SUD treatment professionals who work with women who use MOUD. To be eligible, these professionals needed to meet the following inclusion criteria: (1) prescribe MOUD to women or otherwise provide care (e.g. case management, counseling) to women using MOUD, (2) work in Pennsylvania, and (3) be age 18 or older. These professionals were recruited through advertisements on social media, chain referrals, and direct calls to certified Opioid Treatment Programs as identified by the Substance Abuse and Mental Health Services Administration.

Table 1 details counts of the interviewees across the three categories, along with counts of the specific roles held by SUD treatment and criminal justice professionals. It also notates the number of interviewees who described parenting and pregnancy-related experiences, as well as demographic descriptives of the subsamples.

**Table 1.** Description of respondents’ roles and demographic descriptives.

Category	Professional role	Count	# Who mentioned parenting	# Who mentioned pregnancy	Gender (N/% female)	Age in years (μ/SD)	Race (N/%)				Have children (N/%)
							African-American	Caucasian	Biracial	Not reported	
Women with MOUD experience (N = 20)	—	20	5	5	20/100%	37/7.9	1/5%	14/70%	1/5%	4/20%	14/70%
SUD treatment professionals (N = 12)	MOUD provider	1	1	1	10/83%	48/5	2/17%	8/66%	0/0%	2/17%	—
	Nurse	2	2	0							
	Counselor	3	1	0							
	Case manager/recovery coach	3	2	0							
	Research assistant	1	1	0							
	Treatment program director	2	1	1							
CJ professionals (N = 10)	Treatment court professional	4	2	0	7/70%	44/8	1/10%	9/90%	0/0%	0/0%	—
	Law enforcement	3	0	0							
	State prosecutor	2	0	0							
	Corrections employee	1	0	0							

MOUD: medication for opioid use disorder; SUD: substance use disorder; CJ: criminal justice.

### Interview procedures

After participants were recruited, and their eligibility was confirmed via a brief online survey, study team members contacted eligible participants to schedule phone interviews. Only interested participants were called and scheduled for an interview appointment. No potential participants refused to be interviewed, although scheduling conflicts prevented a few interested participants from scheduling interviews. Each call was one-on-one between the interviewer and the participant. The interviews began with an explanation of the study by trained research staff, who all had undergraduate and graduate-level degrees (BS, MA, and PhD). At the time of the study, all interviewers were employed as research staff and had worked as qualitative research interviewers. All interviewers were women and were from similar demographic backgrounds to the women with MOUD experience; no personal relationship was established between the interviewer and participant prior to the interview, and interviewers did not share personal details about their lives or experiences with the participants.

After explaining the study, participants were asked to provide verbal consent; written consent was not possible, as the interviewers never met participants face-to-face. Following the consent process, interviews were conducted, lasting about an hour each. Interviews were semi-structured, and focused on women’s personal experiences with MOUD treatment or the professionals’ experiences working with women receiving MOUD treatment. The interview questions were not specific to parenting and pregnancy-related experiences; rather, they focused on barriers to accessing MOUD generally and barriers specific to women. Yet, parenting and pregnancy-related barriers were prominent themes that emerged. Participants were compensated with a $50 gift card.

### Data analysis/statistical analysis

All interviews were deidentified and transcribed, with participants assigned unique pseudonyms to protect confidentiality. Transcripts were then coded by study team members in a thematic analysis using *NVivo*. Three study team members independently read through transcripts and generated a list of primary codes; they continued to read additional transcripts until no new codes were added to the codebook. They then repeated this process to generate a list of secondary codes within each primary code. This inductive process of code generation suggested that thematic saturation had been reached. A separate group of four study team members then coded the transcripts using the codebook. Each transcript was coded primarily by one team member. The principal investigator (PI) then double-checked all coded transcripts for consistency and identified discrepancies were discussed and resolved.

## Results

Three subthemes were identified regarding child-rearing as a barrier to initiation and retention in MOUD treatment programs. These are (1) insufficient access to childcare to navigate appointments and meetings, (2) fear of losing custody of, or access to, one’s children, and (3) prioritizing one’s children’s needs before one’s own. The first two subthemes are systemic barriers that can prevent women from accessing MOUD treatment. In contrast, the third subtheme is an internal barrier that can prevent women from utilizing available treatment. In addition, three subthemes were identified concerning pregnancy as a barrier to accessing MOUD treatment. These are (1) hesitancy among physicians to prescribe MOUD for pregnant patients, (2) limited access to resources in rural areas, and (3) difficulty navigating a complex, decentralized health system. Table 2 lists these barriers, as well as representative quotes. All subthemes are described further, and examples are provided below.

**Table 2.** Themes among barriers and representative quotes.

Category	Barrier	Representative quote(s)
Parenting-related Barriers	Lack of stable childcare	“I wish that . . . there were more programs for my kids to do stuff closer to me, because I don’t have childcare. That’s a big issue for me. I can’t get childcare until I work, but I can’t work because I don’t have childcare.”—Woman in Active Recovery “I can name the biggest challenge off the top of my head is childcare. When a woman has children, it is extremely hard for her to seek the treatment that she needs, especially if it’s in-patient, because she doesn’t have support to help her with the children while she’s away.”—SUD Treatment Professional
	Fear of losing custody of/access to children	“That parent wants to go into treatment, and the treatment might take a year or so. Where are those children going to go where it’s safe and they can come back to their mom if mom gets clean? Foster care is like—I don’t know anybody who wants to send their kids to foster care, that’s a caring mom.”—SUD Treatment Professional
	Putting others’ needs before their own	“We’ve seen mothers just run ragged, worried about their kids, trying to help them, and we’re worried about [the mothers’] own health. [We] try to remind them that they’ve got to take care of themselves otherwise and put their sobriety first, the recovery first, so they can be a good mother, which goes against the mother’s nature. They just want to do everything for their kids.”—Criminal Justice Professional “A lot of times women don’t put themselves first or as a priority or think that they are deserving of taking the time to focus on themselves, whether they’re a wife or they have children.”—SUD Treatment Professional
Pregnancy-related barriers	Hesitancy among physicians to prescribe	“I know our doctor here, he won’t treat—he doesn’t see pregnant patients. If I have a female in my caseload on Suboxone maintenance and she becomes pregnant, I’ve got to transfer her out. She’s not coming here anymore because he just doesn’t see pregnant patients. Some doctors are not comfortable prescribing this medication to a pregnant female, I guess.”—SUD Treatment Professional
	Limited resources in rural areas	“We have women here—if they’re pregnant, we have a heck of a time placing them [with appropriate treatment]. If they’re pregnant and are [actively] using [substances], it makes it even worse.”—SUD Treatment Professional
	Difficulty navigating multiple facets of the healthcare system	“. . . the pregnant women that are coming in, a lot of them, they don’t have a lot of experience navigating a health system. It can become very overwhelming, especially with all of the appointments that they have to attend while pregnant. Then, also, for [navigating all the appointments for] the babies when the babies are born too.”—SUD Treatment Professional

SUD: substance use disorder.

### Child rearing as a barrier to receiving MOUD

Our interviews asked about barriers to receiving MOUD treatment, emphasizing gender-specific barriers women face. A common theme mentioned by women with OUD, SUD treatment professionals, and criminal justice professionals alike was the challenges faced by women due to their roles as mothers. Interviewees expressed that child-rearing could impact mothers’ willingness to seek treatment and their ability to remain in treatment once they had begun.

#### Lack of stable childcare

The most pervasive barrier, described by both SUD treatment professionals, criminal justice professionals, and mothers, was the systemic lack of childcare access. While this study intended to focus on MOUD utilization, many interviewees used MOUD in conjunction with other types of OUD treatment, such as in-patient treatment. Having childcare responsibilities decreased access to MOUD across different treatment settings, from MOUD clinics to in-patient treatment facilities.

Mothers often described having difficulty finding MOUD treatment facilities that allowed them to bring their children with them. This posed a challenge, particularly for women who do not have stable access to childcare. One woman who was presently using MOUD succinctly stated, “I wish that . . . there were more programs for my kids to do stuff closer to me, because I don’t have childcare. That’s a big issue for me. I can’t get childcare until I work, but I can’t work because I don’t have childcare.” As a stay-at-home mom, this interviewee did not have access to childcare assistance, but childcare assistance would have helped her better navigate her numerous MOUD appointments.

SUD treatment professionals and criminal justice professionals also acknowledged that the financial barriers posed by childcare needs could create a barrier to accessing treatment, particularly for in-patient treatment and other time-intensive appointments and meetings. One state prosecutor explained:

I often think childcare, if the woman has children, that it becomes a very big challenge for [the woman] to pursue treatment. Often, at the depths of treatments that they need is sometimes in-patient treatment or intensive outpatient treatment that requires a lot of time. That time isn’t afforded to them because they also can’t afford to have childcare during that time.

Women often described how accessing MOUD did not only require them to attend their medical appointments but also to attend accompanying counseling appointments in order to receive their MOUD prescriptions. This would mean having to make multiple appointments weekly, during which they would have to find someone to watch their children if they did not have financial or geographic access to childcare options.

Multiple interviewees noted that childcare is especially a barrier to participation in in-patient residential treatment programs, because getting a spot at a program that allows children can be very difficult. One nurse who works in an outpatient program for pregnant and recently postpartum women succinctly stated:

I can name the biggest challenge off the top of my head is childcare. When a woman has children, it is extremely hard for her to seek the treatment that she needs, especially if it’s in-patient, because she doesn’t have support to help her with the children while she’s away.

Another treatment professional, a counselor in a residential treatment program, similarly noted:

If they come in here and they have children, they need to have appropriate care set up for their kids, and that’s difficult . . . There are few facilities that allow women and children, but again, not everybody wants to take their kid to treatment either.

As these two professionals point out, on one hand, few in-patient facilities allow women to bring their children. However, on the other hand, treatment facilities are often not built or structured to be the type of environment to which mothers may want to bring their children, even if childcare support were available at the facility.

The state prosecutor mentioned above noted that the challenge of accessing childcare specifically impacts female defendants above and beyond those who are men. She explained:

To my knowledge, at least in the treatment facilities that I’ve personally been involved with sending people to, or family members to, or defendants, [the facilities] don’t offer any sort of childcare during the day for people to attend. I hate to be stereotypical, but I think most of the time, the burden of taking care of children falls on the woman. I think that without having some access to affordable childcare, they’re unable to pursue the treatment that they might need.

In her view, because mothers continue to take on the bulk of child-rearing duties, even in two-parent households (including two-parent households where both parents work), lack of childcare prohibits mothers from accessing and staying in treatment, to a level not seen among fathers.

One woman in MOUD treatment suggested that she would have liked to attend an in-patient treatment facility where her children could be involved in her treatment, and she believed her entire family would have benefited from such a treatment option. She told us,

I think that there should be like maybe a place where you have your kids at night and you do your treatment during the day. You involve your children because your children see you use [substances], they should be able to see [you] recover also. Of course, there should be . . . certain stipulations but absolutely [involving children would be useful].

This woman not only wished that there were more facilities that allow women to bring their children, but she also desired the program to concertedly design ways for children to be meaningfully involved in the process.

Another woman we spoke to was lucky enough to find a spot in an in-patient treatment facility where she could bring her children to live with her. However, she described that no formal childcare was provided during her appointments. Hence, she was not able to focus on her treatment as much as she would have preferred:

[Treatment with children] was a little overwhelming because it didn’t really give me time to focus on myself. I get what they were trying to do was like parenting and getting clean at the same time, but it was a little overwhelming because I wasn’t used to really doing that . . . I can’t parent if I’m not all the way 100% by myself. It was like I had to focus on the kids and attempt to attend [group meetings] and stuff. It was a lot.

While being able to bring her children to live at the facility removed barriers to accessing treatment, more resources to help care for her children during appointments would have made the treatment process smoother for her.

In summation, SUD treatment professionals, criminal justice professionals, and women with OUD frequently mentioned lack of access to stable childcare—whether due to financial limitations or the lack of in-patient facilities that accept children—as a crucial barrier to engaging in MOUD treatment.

#### Fear of losing custody of children

Both professionals and women with OUD mentioned that fear of losing custody of their children acted as a systemic barrier for mothers engaging in and completing needed MOUD treatment. On one hand, fear of sparking child welfare involvement alone could prevent women from disclosing their opioid use to a provider and asking for MOUD. On the other hand, seeking more intensive OUD treatment—like in-patient treatment—in conjunction with using MOUD would require that mothers temporarily leave their children with a family member or allow their children to enter foster care, which mothers could fear would lead to a more permanent custody shift.

One SUD treatment professional, a psychiatric nurse who had worked at an in-patient treatment facility, explained that women’s engagement with MOUD treatment is limited by their fear that admitting to a substance use issue could result in child welfare involvement and, potentially, child removal. She told us, “I’m sure that a lot of women are using and will not admit it because they don’t want their kids to go to foster care.” Similar to the comments we shared in the previous section, this nurse felt that this fear could be diminished with greater opportunities for seeking treatment in a supervised, in-patient environment with one’s children. She continued,

I don’t know if we can ever come to that decision on how to handle [patients with children] unless you attach schools or something to live in—dormitories for kids—to the houses of where the women are for treatment.

This psychiatric nurse also observed that MOUD patients who desired in-patient treatment but did not have access to someone who could watch their children, like a family member, may fear their children being placed into foster care. Such fear, she felt, could deter these women from seeking the level of treatment they desired. She explained,

That parent wants to go into treatment, and the treatment might take a year or so. Where are those children going to go where it’s safe and they can come back to their mom if mom gets clean? Foster care is like—I don’t know anybody who wants to send their kids to foster care, that’s a caring mom.

This interviewee emphasized that avoiding treatment to prevent children from entering foster care was indeed a form of maternal care and concern. She sympathized with women who have become stuck with this impossible choice.

Yet, fear of losing custody was also a factor for women who *did* have options beyond foster care. One mother described that she had, at one point, stopped using heroin by beginning a methadone program. Yet she still had the desire to attend an in-patient program, as she felt that receiving counseling and other supports would aid her ability to find stability within her new methadone regimen. However, she was hesitant to leave her children with her own mother, for fear of her mother ultimately gaining permanent custody of them. Ultimately, she told her mother about her desire to enter in-patient treatment, and her mother did indeed use that evidence against her in custody hearings. She told us,

I could still lose [my children]. It would be real easy for me to relapse, but I couldn’t take them [to treatment] because my mum had grandparent rights to my daughter . . . I had to basically ask her permission to go to rehab.

Insofar as mothers must leave their children behind to enter in-patient treatment, fear that leaving children temporarily could lead to a more permanent loss can deter voluntary participation. Nonetheless, *not* seeking treatment voluntarily can ultimately lead to loss of custody anyway and potentially result in court-mandated participation in treatment. The aforementioned nurse, who works in an outpatient program for pregnant and recently postpartum women, told us about an MOUD patient who wanted to participate in more intensive treatment but did not have someone to watch her child. This patient subsequently relapsed and ended up getting arrested. The nurse told us, “The baby was put into foster care. She had to go to jail and then to treatment. She’s still working on getting the baby back. Maybe had she had that childcare, it wouldn’t have played out that way.” This patient was now attempting to succeed in treatment while also having to deal with separation from her child. The interviewee suggested that having the flexibility to attend in-patient or intensive outpatient treatment *before* child separation may have prevented child welfare involvement in the first place.

#### Putting others’ needs before their own

A final theme described by both SUD treatment professionals and criminal justice professionals was an internal experience; they asserted that mothers could feel uncomfortable or worried about prioritizing their own needs, especially at the expense of the perceived needs of their children and family. This discomfort extended to taking time and effort to pursue OUD treatment, even when mothers believed that MOUD treatment would be best for their family in the long term.

One criminal justice professional, a treatment court coordinator, described to us the difficulties she faces trying to convince mothers to focus on their recovery. She explained that some of the largest barriers the women in her program face in succeeding are “issues with being a mother, being a working mother, [and] being a single mother.” She told us,

[We have to] remind them, too, that they got to take care of themselves so they can take care of the kids. We’ve seen mothers just run ragged, worried about their kids, trying to help them, and we’re worried about [the mothers’] own health. [We] try to remind them that they’ve got to take care of themselves otherwise and put their sobriety first, the recovery first, so they can be a good mother, which goes against the mother’s nature. They just want to do everything for their kids.

An SUD treatment professional, a case manager at a methadone clinic, succinctly stated, “A lot of times women don’t put themselves first or as a priority or think that they are deserving of taking the time to focus on themselves, whether they’re a wife or they have children.” As these professionals described, OUD treatment requires a significant time investment. Still, they often found that women could not set this time aside or fully commit themselves, as they were too concerned about their children’s well-being—especially when their children could not be with them in the treatment facility.

While two professionals talked about women putting others’ needs before their own, importantly, none of the mothers we spoke to verbalized this feeling themselves. This is likely because mothers did not see their children’s needs as challenging or a barrier. Mothers fulfilling their children’s needs first may simply be a given, highlighting the importance of adequately creating the treatment environments that interviewees described in the two sections of this paper above. Creating safe and welcoming environments for mothers and their children could allow mothers to address their own needs and their children’s needs simultaneously.

### Pregnancy as a barrier to receiving MOUD

Many of our interviewees were mothers; therefore, those barriers experienced due to motherhood came up quite often when interviewees were asked about the challenges they faced accessing and succeeding in treatment. In comparison to motherhood generally, a smaller number of interviewees identified issues related to pregnancy, as fewer interviewees had experienced having an OUD and seeking treatment while pregnant.

#### Perceived hesitancy by physicians

Whether overtly or covertly, some physicians continue to hesitate or refuse to provide either methadone or buprenorphine to pregnant patients. This hesitancy then, in turn, can lead court system staff members to be reticent to refer pregnant women to those physicians and can lead pregnant patients to feel judged or misunderstood. One individual who works at a treatment program that provides MOUD (who also receives MOUD herself) told us that the physician on staff will not prescribe to pregnant women. Pregnant women who seek services at the clinic are turned away. She explained,

I know our doctor here, he won’t treat—he doesn’t see pregnant patients. If I have a female in my caseload on Suboxone maintenance and she becomes pregnant, I’ve got to transfer her out. She’s not coming here anymore because he just doesn’t see pregnant patients. Some doctors are not comfortable prescribing this medication to a pregnant female, I guess.

This professional knew this norm of rejecting pregnant women was also not constrained to her clinic. She had been told by some of her own clients that similar practices occurred elsewhere. For example, she told us,

This one guy in my caseload, he told me that his cousin is just super duper pregnant right now, and she’s staying with him. She’s got no place to live. She’s shooting heroin every day. He claims that her doctor told her to continue using because she’d jeopardized the baby if she were to quit . . . I was just surprised that he didn’t try to transition her to methadone or something like that.

This professional felt that this hesitancy arose from a lack of understanding by medical professionals of best practices for pregnant women who use drugs. Providers’ hesitancies meant that pregnant women would either have to search for a physician who would prescribe them MOUD or give up and continue to use illicit opioids.

#### Limited resources in rural areas

Interviewees confirmed that finding access to both MOUD and accompanying treatment modalities for pregnant women was even more difficult in rural areas than urban. One SUD treatment professional, an executive director of a recovery support program in a rural area, told us, “We have women here—if they’re pregnant, we have a heck of a time placing them [with appropriate treatment]. If they’re pregnant and are [actively] using [substances], it makes it even worse.”

#### Navigating multiple facets of the healthcare system

A final pregnancy-related barrier described by professionals is that being pregnant and using substances often requires that women receive care from multiple different providers. For women dealing with OUD and accompanying structural vulnerabilities, like housing instability and financial insecurity, navigating even one provider and one set of appointments can be challenging. Seeking care simultaneously for both pregnancy and OUD compounds these challenges. A research assistant and supervisor at an integrated care treatment program relayed,

. . . the pregnant women that are coming in, a lot of them, they don’t have a lot of experience navigating a health system. It can become very overwhelming, especially with all of the appointments that they have to attend while pregnant. Then, also, for [navigating all the appointments for] the babies when the babies are born too.

This same professional described that obstetrics care providers often refer patients to a separate clinic to receive MOUD rather than providing MOUD through their practice. Because prenatal, postnatal, neonatal, and SUD care are typically carried out through separate practices, navigating multiple and varied systems becomes more difficult. Integrating these services into a singular practice could aid pregnant women’s ability to access MOUD while caring for their pregnancies and newborns.

## Discussion

Extant research has documented the unique barriers faced by women in initiating and sustaining treatment for SUDs^16,31^ often related to pregnancy and parenting.^20,22,32,33^ This study adds to prior work by assessing the pregnancy and parenting-related barriers women face in initiating and receiving MOUD as a treatment for OUD specifically, with the goal of elucidating opportunities for improvement in MOUD treatment provision. These barriers are important to assess, especially considering that opioid-related overdose deaths for this population are increasing at a very fast rate.^23^

Six themes emerged from interviews with women who use(d) MOUD, SUD treatment professionals, and criminal justice professionals. Assessing perspectives from these multiple groups allowed for the identification of themes that both converged and diverged across those different perspectives and roles. For example, systemic barriers were a focal point of the women’s responses, while only providers spoke about internal barriers. The first three themes related to parenting: (1) insufficient access to childcare to navigate appointments and meetings; (2) fear of losing custody of, or access to, one’s children; and (3) prioritizing one’s children’s needs before one’s own. The latter three themes were related to pregnancy: (1) hesitancy among physicians to prescribe MOUD for pregnant patients; (2) limited access to resources in rural areas; and (3) difficulty navigating a complex, decentralized healthcare system. These themes are similar to those barriers identified in previous work, for SUD treatment more generally.^20^ These themes also suggest that there are systemic changes that could be made to treatment programs to reduce the barriers that pregnant and parenting women face in accessing MOUD treatment.

Many women with OUD are hesitant to engage in programs that provide MOUD—especially time-intensive and residential treatment programs—that would require them to be away from their children. As was described in the introduction, only 5.5% of all American SUD treatment facilities provide childcare.^19^ Additional childcare support is needed at both in-patient and outpatient treatment programs to assuage women’s barriers related to childcare, as well as the fear of losing access to their children if they spend time away from their children for treatment.

Women’s fear of losing custody of their children is well-founded. Substance use is associated with child maltreatment reports generally^34^ as well as more complex and severe reports of maltreatment.^35^ As would then be expected, child welfare caseloads are positively associated with substance use rates across most counties; this is true even when controlling for demographic and socioeconomic factors.^35^ These associations may be mitigated if both the child welfare system and criminal justice system worked together to promote and prioritize parental access to SUD treatment. Having less punitive access to treatment would perhaps then lessen mothers’ fear of losing custody of their children, as well as lessen instances of child maltreatment.

Access to comprehensive, integrated care is another systemic improvement that may reduce barriers for women. Interviewees described that it can be difficult for pregnant and postpartum women to navigate their many appointments, such as MOUD and other treatment-related appointments and obstetric and pediatric appointments. Integrated substance use treatment programs have been associated with improved outcomes for mothers with SUDs, as well as their children, when compared to non-integrated programs. Mothers engaged in integrated programs attend a higher number of prenatal appointments, have a lower likelihood of premature birth, endure shorter infant hospitalization times, and have fewer positive urine toxicology tests at the time of delivery.^36,37^

Access to appropriate treatment is a particular concern for women living in rural areas. In rural communities, a limited number of clinics prescribe MOUD and there are limited resources to support people receiving MOUD. Rural communities can also be quite geographically distant from one other, meaning that getting to clinics and resources in other towns, especially without public transportation infrastructure, can be difficult. Hence, substance use treatment and recovery resources are more difficult to access in rural areas than in urban areas.^38^ Furthermore, because substance use stigma and misunderstanding are particularly pronounced in rural areas,^39^ the chance that existing clinics that may turn away pregnant women could be especially high. Increased access to telehealth may be a viable solution to this concern, as telehealth has been associated with increased MOUD access and increased patient satisfaction with MOUD services.^40^

A notable strength of this qualitative study is the triangulation of multiple perspectives on barriers to MOUD treatment, including women who have used MOUD and criminal justice and SUD treatment professionals who work with women who use MOUD. There are, however, limitations to the study design. One of these is that the study team cannot verify participants’ experiences. It is important to note that regardless of verification, participants’ perspectives and perceptions are still meaningful to them and influence their behavior; these perspectives can still greatly contribute to understanding pregnancy- and parenting-related barriers women face regarding MOUD initiation and retention. Another limitation is that interviewees were recruited from a single state in the Northeastern United States, and thus, there is limited representation across age, race/ethnicity, and geography. Given that women of color are disproportionately impacted by the child welfare system, concerns about childcare and child custody loss may be especially strong for this population.^41^ Future studies should assess the experiences of a more diverse group of women, particularly women of color, from multiple locations across the country. Finally, very few women described pregnancy-related barriers. This could have been a limitation of the semi-structured interview questions, as there were no questions that directly asked participants about being pregnant while seeking MOUD treatment—any references to pregnancy came from the women organically mentioning it in their responses. This also could have been a limitation of our sample; while most women had children, none were presently pregnant, and therefore, the salient barriers of pregnancy may have been less present in their treatment and recovery stories at the timepoint of the interview. Nonetheless, while there is a need for more research specific to this area, these interviewees still importantly described how pregnancy can act as a barrier to receiving MOUD.

## Conclusion

In summation, the barriers to initiating and continuing MOUD described by treatment-seekers and professionals are very similar to the barriers experienced by pregnant and parenting women when seeking SUD treatment, generally. In response to these barriers, we recommend two specific calls to action: First, improved affordable childcare infrastructure not only in the United States broadly, but also at MOUD clinics and other SUD treatment programs specifically. Second, more integrated care programs are needed, at which pregnant and recently postpartum women may seek not only MOUD treatment but also obstetric, therapeutic, and pediatric care for themselves and their infants. Improving access to MOUD for pregnant and parenting women is an opportunity to improve both mothers’ OUD recovery-related outcomes and fetuses’ and children’s health and well-being.

## Declarations

**Ethics approval and consent to participate:** This work was approved by the Pennsylvania State University Institutional Review Board (STUDY00018974) between May and August 2022. All participants provided verbal consent before their interview. Written consent was not possible, as the interviewers never met participants face-to-face. Interviews followed the consent process, lasting about an hour each.

**Consent for publication:** Informed consent for publication was provided by the participants.

**Availability of data and materials:** These data are not publicly available. Data may be made available upon reasonable request.
